# Sarcopenia, an independent predictor for all-cause mortality in rheumatoid arthritis: Insights from the NHANES database

**DOI:** 10.1097/MD.0000000000050000

**Published:** 2026-07-31

**Authors:** Xiaodong Zhang, Hua Liu, Tianzuo Lan, Xin Cai

**Affiliations:** a Department of Rheumatology and Immunology, The First People’s Hospital of Guiyang, Guiyang, Guizhou, China; b Urology, The Second Affiliated Hospital of Guizhou University of Chinese Medicine, Guiyang, Guizhou, China.

**Keywords:** mortality, NHANES, rheumatoid arthritis, sarcopenia, skeletal muscle mass

## Abstract

Sarcopenia, characterized by a decline in muscle mass and function, is increasingly recognized as a significant comorbidity in chronic diseases, including rheumatoid arthritis (RA). However, its relevance to all-cause mortality in RA patients remains to be explored. Here, we examine the correlation between sarcopenia and all-cause mortality in RA patients using the NHANES database. Data were obtained from 910 RA patients identified through self-reported data in the nationwide NHANES (1999–2018). Sarcopenia was defined by an appendicular skeletal muscle mass index adjusted for body mass index (ASM/BMI). Cox regression models were utilized to measure the connection between sarcopenia and all-cause mortality, adjusted for demographic, clinical, and lifestyle factors. Moreover, subgroup and sensitivity analyses were also performed. Sarcopenia was independently linked to a higher risk of all-cause mortality (HR = 1.610, 95% CI: 1.192–2.176, *P* = .002) after adjusting for potential confounders. Subgroup analysis showed significant associations in individuals aged <60 years, males, and those with comorbid conditions such as hypertension, diabetes, and cardiovascular disease. Additionally, sensitivity analysis confirmed the robustness of the findings. Sarcopenia was associated with a higher mortality risk in patients with RA, suggesting that its early identification and management may improve survival outcomes.

## 1. Introduction

Rheumatoid arthritis (RA) is a systemic autoimmune disorder characterized by chronic inflammation that leads to progressive organ damage. Despite significant therapeutic advances over the past decades, including the widespread use of disease-modifying antirheumatic drugs and targeted therapies, patients with RA continue to face a significantly higher all-cause mortality than the general population.^[1-3]^ This enduring mortality gap is quantified by standardized mortality ratios reported between 1.4 and 2.1 in large-scale cohort studies.^[4-6]^

Sarcopenia is defined by the Foundation for the National Institutes of Health (FNIH) Sarcopenia Project as the combination of low lean mass and muscle weakness associated with disability, frailty, and all-cause mortality.^[7,8]^ Sarcopenia affects not only the elderly but also individuals with chronic inflammatory diseases, where systemic inflammation, metabolic disruption, and reduced physical activity contribute to muscle degradation.^[9,10]^ Previous investigations indicate that the estimated prevalence of sarcopenia in the RA population ranges between 20% and 40%.^[11,12]^ This variability may be partly attributable to differences in the diagnostic criteria used across studies. Specifically, these criteria differ in the emphasis placed on low muscle mass, low muscle strength, and physical performance, as well as in the indices and cutoff values used for diagnosis.^[12]^ The increased susceptibility of patients with RA to sarcopenia, compared with the general population, is linked to inactivity associated with the disease, metabolic disruptions, and long-term corticosteroid use.^[13,14]^ Consequently, the presence of sarcopenia in the RA population is clearly linked to greater functional impairment and a higher burden of comorbidities.^[15]^

Sarcopenia is a well-established predictor of all-cause mortality across various populations, including the elderly and individuals with chronic conditions like cancer and cardiovascular disease.^[16-18]^ In the context of RA, it is a frequent comorbidity known to exacerbate functional impairment and increase the likelihood of adverse events such as fractures and falls.^[19-21]^ This vulnerability is often compounded by the common use of corticosteroids in RA management, which can accelerate muscle degradation.^[20]^ However, despite the well-documented association between sarcopenia and these adverse physical outcomes in RA, its specific contribution to the mortality burden in this population remains poorly understood.

Therefore, the present study was designed to address this critical gap by investigating the association between sarcopenia and all-cause mortality in a large cohort of individuals with RA. By clarifying the prognostic significance of sarcopenia, these findings may help enhance risk stratification and inform the development of future clinical management strategies for this vulnerable patient population.

## 2. Materials and methods

### 2.1. Study populations

**Figure 1.**
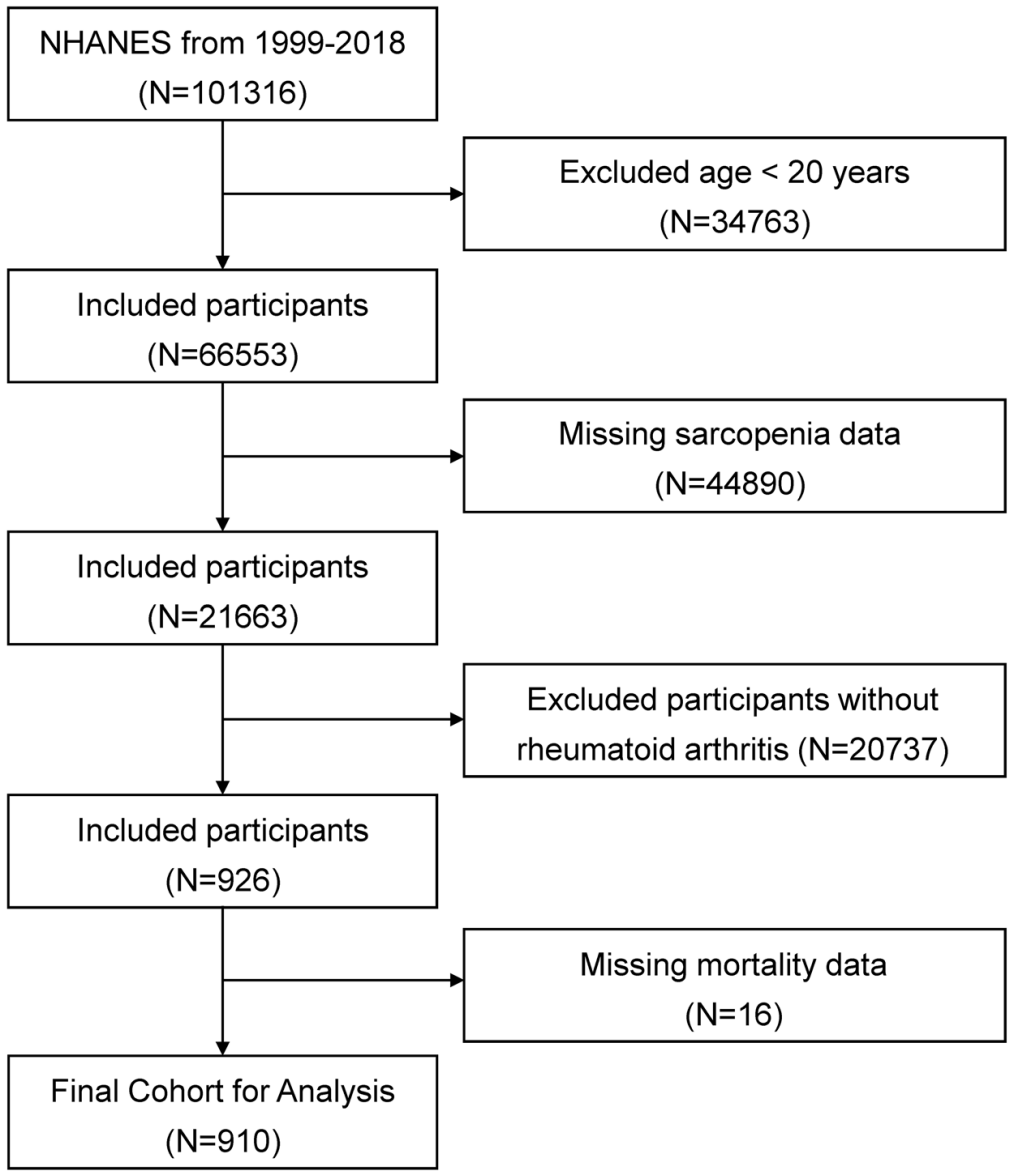
Flow chart. NHANES = National Health and Nutrition Examination Survey.

This study obtained data from the National Health and Nutrition Examination Survey (NHANES), a nationwide program monitoring health and nutritional status through structured questionnaires, physical examinations, and laboratory tests.^[22]^ All protocols were approved by the National Center for Health Statistics Ethics Review Board, and written informed consent was obtained from all participants. Data from ten consecutive cycles (1999–2018) were analyzed. Of the initial 101,316 participants, 34,763 individuals under 20 years old were excluded. Moreover, 20,737 individuals without RA status and 44,890 individuals with missing sarcopenia data were excluded. Finally, 16 individuals without complete mortality data were removed, resulting in a final cohort of 910 participants (Fig. 1).

### 2.2. RA population

Rheumatoid arthritis was determined through self-reported responses based on an interview, with similar efficiency compared to clinical assessments confirmed by previous investigations.^[23,24]^ Participants were first asked whether they had ever been diagnosed with any form of arthritis by a healthcare professional. Those who answered affirmatively were then prompted to specify the type, with some individuals being diagnosed with rheumatoid arthritis.

### 2.3. Definition of sarcopenia

Sarcopenia was identified using appendicular skeletal muscle mass (ASM)/body mass index (BMI). ASM was calculated by adding lean soft tissue from both upper and lower limbs, which was quantified through dual-energy x-ray absorptiometry (DXA). In NHANES, whole-body DXA scans were performed using Hologic QDR 4500A fan-beam densitometers in 1999 to 2006 and Hologic Discovery A densitometers in 2011 to 2018 according to standardized procedures. Examinations were conducted by certified radiology technologists with routine quality-control measures, including phantom scans and centralized review. To account for inter-individual variation in body composition, ASM was standardized by dividing BMI, producing a relative muscle index. Following diagnostic thresholds established by the FNIH, individuals with an ASM/BMI ratio below 0.789 for men or 0.512 for women were classified as having sarcopenia.^[25,26]^ Determination of sarcopenia status focused on participants with an affirmative RA diagnosis and available DXA records. Ineligibility for DXA examination was associated with pregnancy or body dimensions exceeding the scanner limits of 192.5 cm in height or 136.4 kg in weight.^[27]^

### 2.4. Outcome ascertainment

Mortality status was determined from records of the National Death Index, a comprehensive vital status database maintained by the National Center for Health Statistics (NCHS). The follow-up period was calculated from the date of enrollment to the date of death or December 31, 2019, whichever occurred first. Participants who did not meet NCHS criteria for determining mortality status were excluded from the analysis.^[28]^ This study focused exclusively on all-cause mortality, without further classification by specific causes of death.

### 2.5. Assessment of covariates

Covariates included age, sex, ethnicity, education level, marital status, poverty-to-income ratio (PIR), smoking status, alcohol intake, physical activity, BMI, hypertension, hyperlipidemia, diabetes mellitus, cardiovascular disease, HbA1c, total cholesterol, and uric acid, all defined following NHANES protocols.^[29]^ Demographic and lifestyle factors were collected via structured interviews. Physical activity was assessed using the NHANES physical activity questionnaire and quantified as metabolic equivalent of task (MET)-min/wk based on activity type, weekly frequency, and duration. Comorbidities were identified through a combination of self-reported diagnoses, medication use, clinical evaluation, and laboratory tests. Hypertension was diagnosed by a history of diagnosis, antihypertensive medication use, or blood pressure ≥ 140/90 mm Hg. Hyperlipidemia was based on diagnosis, lipid-lowering treatment, or abnormal cholesterol levels. Diabetes mellitus was determined by reported history, antidiabetic medication use, fasting glucose ≥ 126 mg/dL, or HbA1c ≥ 6.5%. Laboratory indicators were obtained using standardized NHANES assay procedures.

### 2.6. Statistical analysis

Baseline characteristics were compared between participants with and without sarcopenia. Continuous variables were presented as means with standard deviations or medians with interquartile ranges and were compared using the *t* test or Wilcoxon test, as appropriate. Categorical variables were presented as counts and percentages and compared using chi-square or Fisher test. Three cox regression models were utilized, with results shown in hazard ratio (HR) and 95% confidence interval (CI). The proportional hazards assumption was evaluated using Schoenfeld residuals, and no violation was detected (Fig. S1, Supplemental Digital Content 1). Model 1 was unadjusted; model 2 was adjusted for demographic factors; model 3 included additional adjustments for BMI, smoking status, alcohol intake, hypertension, hyperlipidemia, diabetes mellitus, cardiovascular disease, total cholesterol, HbA1c and serum uric acid. Survival analysis with the log-rank test was carried out to compare all-cause mortality between sarcopenic and non-sarcopenic participants. Subgroup analyses were conducted to assess the consistency of the association between sarcopenia and mortality across demographic and clinical strata based on model 3. Additionally, interaction terms were tested to assess effect modification. Missing values in categorical covariates were handled by coding missingness as an additional category, allowing all participants to be included in the primary analysis. Sensitivity analyses were performed to verify the robustness of the main findings, including a complete-case analysis and additional analyses in which model 3 was further adjusted for serum 25-hydroxyvitamin D or physical activity (MET-min/wk). All statistical analyses were performed using R software (version 4.4.1; R Foundation for Statistical Computing, Vienna, Austria). Sampling weights were not applied since we focused on internal associations within a defined analytic cohort rather than producing nationally representative estimates. A *P*-value < .05 was considered statistically significant (2-tailed).

## 3. Results

### 3.1. Baseline characteristics

Baseline characteristics of the 910 RA participants stratified by sarcopenia status are shown in Table 1. A total of 180 individuals (19.78%) were identified as having sarcopenia. According to our findings, participants with sarcopenia were significantly older (63.15 vs 55.51 years) and had a lower educational level. Moreover, a higher incidence of diabetes mellitus (40.00% vs 18.36%), cardiovascular disease (33.33% vs 19.86%), hypertension (66.67% vs 56.44%), and hyperlipidemia (86.11% vs 76.03%) was observed. Mexican Americans had the highest prevalence of sarcopenia (44.07%), followed by Other Hispanics (20.37%), Non-Hispanic Whites (18.27%), Other Races (14.29%), and Non-Hispanic Blacks (4.74%). Sarcopenic participants also had higher HbA1c levels (6.12 vs 5.78) and serum uric acid concentrations (5.71 vs 5.43 mg/dL), along with a greater prevalence of obesity (BMI > 30 kg/m^2^: 57.22% vs 37.40%). Additionally, they were identified with lower income levels indicated by PIR and had significant differences in alcohol intake. No significant differences were observed regarding sex, smoking status, total cholesterol, and 25-hydroxyvitamin D (all *P* > .05).

**Table 1:** Baseline characteristics of participants stratified by sarcopenia status.

Characteristics	Total (n = 910)	Sarcopenia		*P* value
		No (n = 730)	Yes (n = 180)	
Age, mean (SD)	57.02 (14.37)	55.51 (14.26)	63.15 (13.19)	<.001
Sex, n (%)				.682
Male	372 (40.88)	296 (40.55)	76 (42.22)	
Female	538 (59.12)	434 (59.45)	104 (57.78)	
Ethnicity, n (%)				<.001
Mexican American	177 (19.45)	99 (13.56)	78 (43.33)	
Other Hispanic	54 (5.93)	43 (5.89)	11 (6.11)	
Non-Hispanic White	405 (44.51)	331 (45.34)	74 (41.11)	
Non-Hispanic Black	232 (25.49)	221 (30.27)	11 (6.11)	
Other race	42 (4.62)	36 (4.93)	6 (3.33)	
Education level, n (%)				<.001
Less than high school	367 (40.33)	271 (37.12)	96 (53.33)	
High school or equivalent	229 (25.16)	182 (24.93)	47 (26.11)	
College or above	314 (34.51)	277 (37.95)	37 (20.56)	
Marital status, n (%)				.005
Married and a partner	508 (55.82)	399 (54.66)	109 (60.56)	
Never married	70 (7.69)	64 (8.77)	6 (3.33)	
Widowed, divorced or separated	316 (34.73)	258 (35.34)	58 (32.22)	
Not recorded	16 (1.76)	9 (1.23)	7 (3.89)	
Poverty to income ratio, n (%)				.011
<1.3	311 (34.18)	240 (32.88)	71 (39.44)	
1.3–3.5	316 (34.73)	251 (34.38)	65 (36.11)	
>3.5	213 (23.41)	187 (25.62)	26 (14.44)	
Not recorded	70 (7.69)	52 (7.12)	18 (10.00)	
Smoking status, n (%)				.138
Never	391 (42.97)	307 (42.05)	84 (46.67)	
Current	262 (28.79)	221 (30.27)	41 (22.78)	
Former	257 (28.24)	202 (27.67)	55 (30.56)	
Alcohol intake, n (%)				.010
Never	393 (43.19)	308 (42.19)	85 (47.22)	
Current	250 (27.47)	217 (29.73)	33 (18.33)	
Former	223 (24.51)	168 (23.01)	55 (30.56)	
Not recorded	44 (4.84)	37 (5.07)	7 (3.89)	
Body mass index, n (%)				<.001
<25	225 (24.73)	208 (28.49)	17 (9.44)	
25–30	309 (33.96)	249 (34.11)	60 (33.33)	
>30	376 (41.32)	273 (37.40)	103 (57.22)	
Hypertension, n (%)				.013
No	378 (41.54)	318 (43.56)	60 (33.33)	
Yes	532 (58.46)	412 (56.44)	120 (66.67)	
Hyperlipidemia, n (%)				.003
No	200 (21.98)	175 (23.97)	25 (13.89)	
Yes	710 (78.02)	555 (76.03)	155 (86.11)	
Diabetes mellitus, n (%)				<.001
No	704 (77.36)	596 (81.64)	108 (60.00)	
Yes	206 (22.64)	134 (18.36)	72 (40.00)	
Cardiovascular disease, n (%)				<.001
No	705 (77.47)	585 (80.14)	120 (66.67)	
Yes	205 (22.53)	145 (19.86)	60 (33.33)	
HbA1c, %, mean (SD)	5.85 (1.13)	5.78 (1.11)	6.12 (1.18)	<.001
Total cholesterol, mmol/L, mean (SD)	5.22 (1.10)	5.21 (1.10)	5.24 (1.12)	.764
Uric acid, mg/dL, mean (SD)	5.49 (1.59)	5.43 (1.60)	5.71 (1.50)	.033
25-hydroxyvitamin D, (nmol/L), mean (SD)	58.85 (22.98)	59.27 (23.45)	56.99 (20.81)	.321
Physical activity, MET-min/wk, median (IQR)	756.00 (252.00–2412.07)	756.00 (252.00–2418.10)	720.00 (252.00–2400.00)	.597

HbA1c = hemoglobin A1c, IQR = interquartile range, MET = metabolic equivalent of task, SD = standard deviation.

### 3.2. Associations of sarcopenia with mortality in RA patients

**Figure 2.**
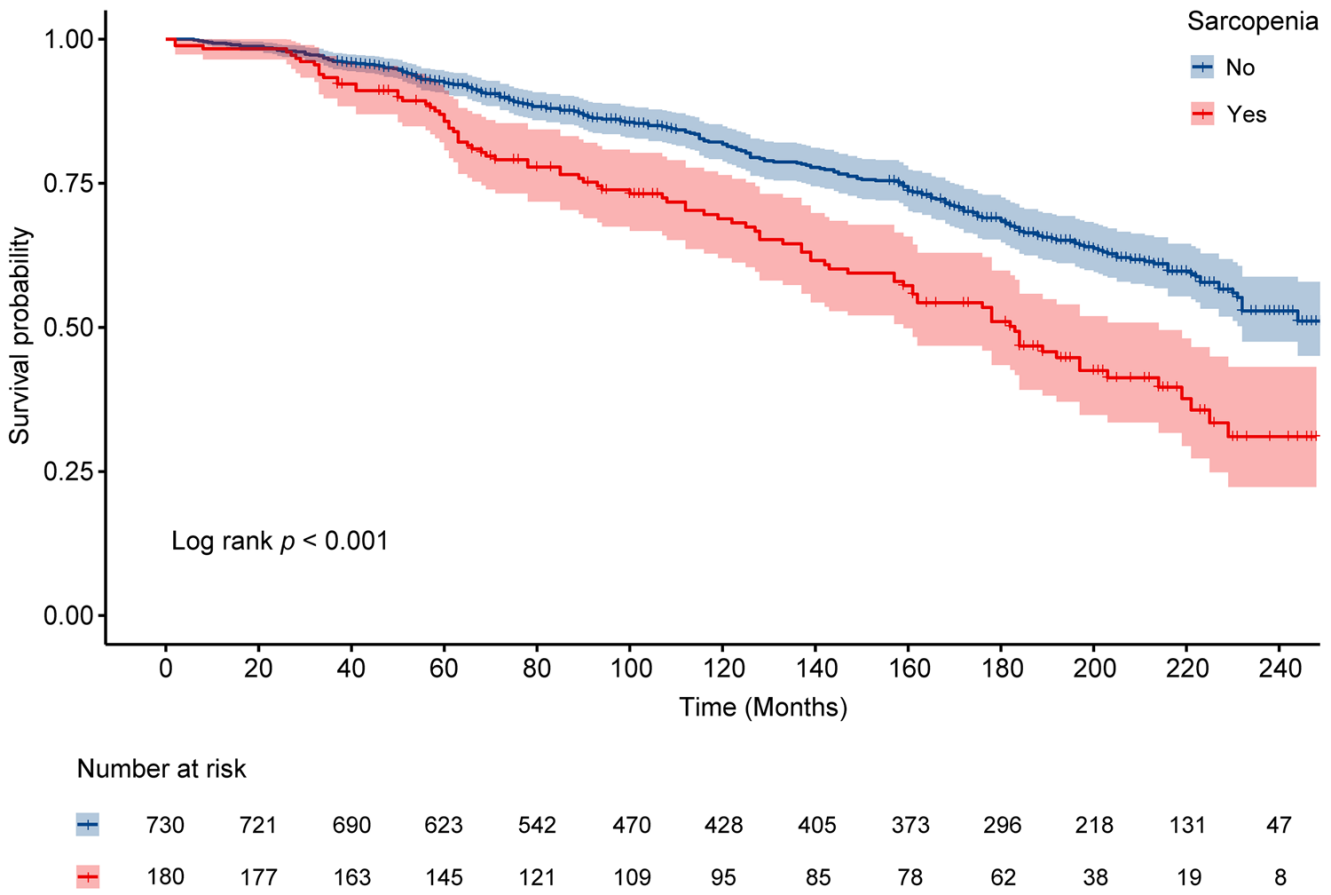
Kaplan–Meier analysis of all-cause mortality stratified by sarcopenia in RA. RA = rheumatoid arthritis.

The correlation of sarcopenia and all-cause mortality in RA patients is presented in Table 2. In unadjusted model 1, sarcopenia was significantly associated with higher mortality risk (HR = 1.864, 95% CI: 1.459–2.381, *P* < .001), which remained in model 2 (HR = 1.604, 95% CI: 1.193–2.158, *P* = .002). In model 3, the association persisted with an HR of 1.610 (95% CI: 1.192–2.176, *P* = .002), indicating the independent correlation of sarcopenia with increased mortality risk. The Kaplan–Meier curves for all-cause mortality can be found in Figure 2, showing a significantly lower survival probability in patients with sarcopenia (log-rank *P* < .001).

**Table 2:** Association of sarcopenia with all-cause mortality in RA patients.

Group	Model 1	*P* value	Model 2	*P* value	Model 3	*P* value
	HR (95% CI)		HR (95% CI)		HR (95% CI)	
Non-sarcopenia	Reference		Reference		Reference	
Sarcopenia	1.864 (1.459–2.381)	<.001	1.604 (1.193–2.158)	.002	1.610 (1.192–2.176)	.002

Model 1: Unadjusted crude model.

Model 2: Adjusted model for age, sex, race/ethnicity, education level, marital status, PIR.

Model 3: Adjusted model incorporating additional adjustments for BMI, smoking, alcohol intake, hypertension, hyperlipidemia, diabetes mellitus, cardiovascular disease, total cholesterol, HbA1c, uric acid.

BMI = body mass index, CI = confidence intervals, HbA1c = hemoglobin A1c, HR = hazard ratio, PIR = poverty-to-income ratio, RA = rheumatoid arthritis.

### 3.3. Subgroup analyses

The correlation was validated in several subgroups, as presented in Table 3. Sarcopenia was significantly associated with a higher mortality risk in patients aged <60 years (HR = 2.312, 95% CI: 1.081–4.946, *P* = .031), but not in the population ≥60 years old (HR = 1.257, 95% CI: 0.897–1.761, *P* = .183). A significantly higher mortality risk was observed in males with sarcopenia (HR = 2.305, 95% CI: 1.463–3.632, *P* < .001), but not in females (HR = 1.283, 95% CI: 0.820–2.009, *P* = .276). Regarding lifestyle and clinical factors, sarcopenia contributed to higher mortality risk in current smokers (HR = 2.596, 95% CI: 1.310–5.145, *P* = .006) and current drinkers (HR = 2.021, 95% CI: 1.282–3.184, *P* = .002), as well as in patients with a BMI between 25 and 30 (HR = 3.307, 95% CI: 1.956–5.592, *P* < .001), hypertension (HR = 2.580, 95% CI: 1.372–4.853, *P* = .003), diabetes mellitus (HR = 2.126, 95% CI: 1.240–3.645, *P* = .006), cardiovascular disease (HR = 1.738, 95% CI: 1.161–2.602, *P* = .007), and hyperlipidemia (HR = 1.449, 95% CI: 1.012–2.073, *P* = .043). No significant interaction was detected between sarcopenia and any of the stratifying variables (all *P* for interaction > .05). All subgroup analyses were performed using model 3, adjusting for relevant covariates while excluding the stratification variable in each specific subgroup.

**Table 3:** Association of sarcopenia with all-cause mortality across subgroups of RA patients.

Subgroups	N	HR (95% CI)	*P* value	*P* interaction
Age				.730
<60	500	2.312 (1.081–4.946)	.031	
≥60	410	1.257 (0.897–1.761)	.183	
Sex				.074
Male	372	2.305 (1.463–3.632)	<.001	
Female	538	1.283 (0.820–2.009)	.276	
Ethnicity				.517
Mexican American	177	1.219 (0.596–2.494)	.587	
Other Hispanic	54	1.274 (0.921–1.762)	.143	
Non-Hispanic White	405	1.496 (0.987–2.268)	.058	
Non-Hispanic Black	232	1.806 (0.577–5.648)	.310	
Other race	42	1.155 (0.846–1.576)	.364	
Education level				.802
Less than high school	367	1.295 (0.811–2.068)	.278	
High school or equivalent	229	1.283 (0.731–2.252)	.385	
College or above	314	1.852 (0.785–4.372)	.160	
Smoking status				.338
Never	391	1.381 (0.837–2.279)	.206	
Current	262	2.596 (1.310–5.145)	.006	
Former	257	1.729 (0.991–3.015)	.054	
Alcohol intake				.480
Never	393	1.050 (0.426–2.587)	.915	
Current	250	2.021 (1.282–3.184)	.002	
Former	223	1.324 (0.758–2.313)	.325	
Body mass index				.125
<25	225	1.006 (0.409–2.475)	.990	
25–30	309	3.307 (1.956–5.592)	<.001	
>30	376	1.380 (0.830–2.295)	.214	
Hypertension				.094
No	378	1.271 (0.876–1.843)	.206	
Yes	532	2.580 (1.372–4.853)	.003	
Hyperlipidemia				.659
No	200	1.447 (0.916–2.285)	.113	
Yes	710	1.449 (1.012–2.073)	.043	
Diabetes mellitus				.155
No	704	1.369 (0.919–2.039)	.122	
Yes	206	2.126 (1.240–3.645)	.006	
Cardiovascular disease				.911
No	705	1.543 (0.912–2.612)	.106	
Yes	205	1.738 (1.161–2.602)	.007	

In subgroup analyses, model 3 was applied while excluding the stratification variable from covariate adjustments within each specific subgroup.

CI = confidence intervals, HR = hazard ratio, RA = rheumatoid arthritis.

### 3.4. Sensitivity analyses

In the sensitivity analysis, baseline characteristics of 758 participants with complete covariate data (Table S1, Supplemental Digital Content 2) were included. Sarcopenia played a significant role in increasing all-cause mortality across all models, consistent with the primary analysis. In the unadjusted model (model 1), the HR for sarcopenia was 1.846 (95% CI: 1.402–2.430, *P* < .001), which adjusted to 1.658 (95% CI: 1.221–2.250, *P* = .001) in model 2, and 1.593 (95% CI: 1.146–2.215, *P* = .006) in model 3 (Table S2, Supplemental Digital Content 3). The association remained significant after additional adjustment for serum 25-hydroxyvitamin D (HR = 1.652, 95% CI: 1.102–2.477, *P* = .015) and physical activity (HR = 1.632, 95% CI: 1.082–2.462, *P* = .019). Survival analysis with Kaplan–Meier curves (Fig. S2, Supplemental Digital Content 4) further confirmed the findings, showing a significantly lower survival probability in sarcopenic patients (log-rank *P* < .001), confirming the correlation observed in the primary analysis.

## 4. Discussion

This study showed an association between sarcopenia and increased all-cause mortality in patients with RA. After adjusting for multiple confounders, sarcopenia was associated with a 61% higher risk of mortality, and this association remained consistent in sensitivity analyses. This finding suggests that muscle health warrants attention in this population and that early management of sarcopenia may be beneficial to long-term survival.

Our finding that sarcopenia is significantly associated with all-cause mortality in patients with RA is consistent with a large body of evidence from diverse populations. For instance, a meta-analysis by Xu et al^[30]^ demonstrated a significant mortality association (HR = 2.00), while another by Westbury et al^[8]^ reported a pooled HR of 2.75, underscoring the consistent relationship between muscle loss and poor outcomes. This link has been further substantiated in various specific conditions, with studies in chronic kidney disease,^[31]^ liver cirrhosis,^[32]^ and type 2 diabetes,^[33]^ all identifying sarcopenia as a critical factor associated with higher mortality. Within the specific context of RA, our results are particularly aligned with key recent studies. Consistent with findings from Xu et al^[34]^ in a smaller RA cohort of 558 patients (adjusted HR = 1.826–2.112), our study in a larger cohort (n = 910) confirmed a significant association with a comparable hazard ratio (adjusted HR = 1.610). Our observed sarcopenia prevalence (19.8%) was also comparable to that reported by Shin et al^[35]^ (20.5%). Our work also builds upon that of Bennett et al,^[9]^ who previously highlighted the link between sarcopenia and physical disability in RA. This is further supported by Wiegmann et al,^[36]^ who found sarcopenia in RA diminishes quality of life, and is often exacerbated by glucocorticoid use, a key factor identified by Yamada et al.^[37]^ Moreover, its synergistic effects with osteoporosis are known to significantly increase fracture likelihood.^[20,38,39]^ While these previous studies established a clear link between sarcopenia and adverse physical outcomes, our longitudinal analysis extends this by demonstrating its significant association with all-cause mortality. Additionally, our subgroup analysis revealed that the strength of this association varies by patient characteristics. Collectively, these findings suggest the potential value of integrating sarcopenia assessment into the clinical management of RA, a strategy that may aid in refining patient stratification and better informing long-term prognosis.

The association between sarcopenia and all-cause mortality in patients with RA appears to be context-dependent, varying by demographic factors such as sex and age. The heightened vulnerability observed in males, a group in whom RA is linked to more pronounced lean mass deficits,^[40]^ may be explained by the pivotal role of testosterone. Low testosterone levels present a dual challenge for these patients: they are not only associated with an increased risk of developing RA itself,^[41]^ but are also fundamental to maintaining muscle mass,^[42]^ partly by mitigating muscle wasting^[43]^ and reducing protein breakdown.^[44]^ In addition to these sex-specific factors, the observation that this association was more pronounced in younger than in older adults is also insightful. This may suggest that in younger individuals, sarcopenia serves as a more direct indicator of a severe underlying disease phenotype, whereas in older adults, its prognostic signal is likely attenuated by the confounding influence of natural, age-related muscle decline.^[45–47]^

The development of sarcopenia in RA is multifactorial, driven by both the inherent inflammatory state of the disease and common therapeutic interventions. Chronic inflammation in RA is a primary driver of muscle degradation. Key inflammatory cytokines, particularly tumor necrosis factor-alpha and interleukin-6, are known to activate proteolytic pathways that lead to muscle wasting.^[48]^ This inflammatory environment may also alter macrophage polarization towards a pro-catabolic M1 phenotype,^[49,50]^ while concurrent metabolic disruptions, including altered lipid metabolism and impaired protein synthesis, further contribute to the loss of muscle mass.^[51,52]^ Compounding these disease-driven processes, glucocorticoid (GC) therapy, a common component of RA management, significantly exacerbates muscle atrophy. GCs are known to directly interfere with muscle health by reducing both mass and function,^[53]^ with higher doses linked to a greater likelihood of muscle loss.^[37]^ Mechanistically, GCs promote muscle degradation by upregulating key catabolic regulators like myostatin and atrogenes, such as atrogin-1 and MuRF-1.^[54]^ The deleterious impact of these therapies is often amplified by vitamin D deficiency, a common issue in patients undergoing GC treatment.^[55]^ These findings, therefore, suggest that sarcopenia may reflect an underlying systemic burden that is also associated with all-cause mortality in patients with RA.

This study has several strengths. First, we used the NHANES database, which provided a large sample size and established a solid foundation for our analysis. Second, our Cox regression models controlled for a wide range of demographic, clinical, and lifestyle indicators, which supports the robustness of the results. Additionally, subgroup and sensitivity analyses were conducted to further validate our findings. However, this study also has several limitations. To begin with, the reliance on self-reported data, such as the RA diagnosis, is a potential source of information bias, even if the method has been previously validated. The assessment of sarcopenia using body composition data could also be influenced by potential measurement errors. Furthermore, our analysis did not account for several important clinical variables, such as disease activity, specific treatment regimens, or nutritional status, and the absence of these data introduces the possibility of residual confounding. Lastly, the retrospective nature of the data limited our ability to control for dynamic changes in disease progression over time. Therefore, the long-term relationship between sarcopenia and mortality in patients with RA needs to be further investigated with prospective studies.

## 5. Conclusion

The present longitudinal analysis identifies an association between sarcopenia and an elevated risk of all-cause mortality among patients with RA. This finding underscores the clinical relevance of monitoring skeletal muscle health in this population. Furthermore, the observed association provides a rationale for future research to evaluate the relationship between muscle status and long-term outcomes.

## Acknowledgments

We gratefully acknowledge the invaluable contributions of all NHANES participants to this research.

## Author contributions

**Data curation:** Xiaodong Zhang, Hua Liu.

**Formal analysis:** Xiaodong Zhang, Hua Liu.

**Investigation:** Xiaodong Zhang, Hua Liu.

**Methodology:** Xiaodong Zhang, Hua Liu.

**Visualization:** Xiaodong Zhang.

**Writing – original draft:** Xiaodong Zhang, Hua Liu.

**Writing – review & editing:** Tianzuo Lan, Xin Cai.








