# Supplementary figures and images for: Sarcopenia, an independent predictor for all-cause mortality in rheumatoid arthritis: Insights from the NHANES database
Source: Medicine (Baltimore). 2026 Jul 31;105(31):e50000. doi: 10.1097/MD.0000000000050000 (PMC13433025; doi:10.1097/MD.0000000000050000)

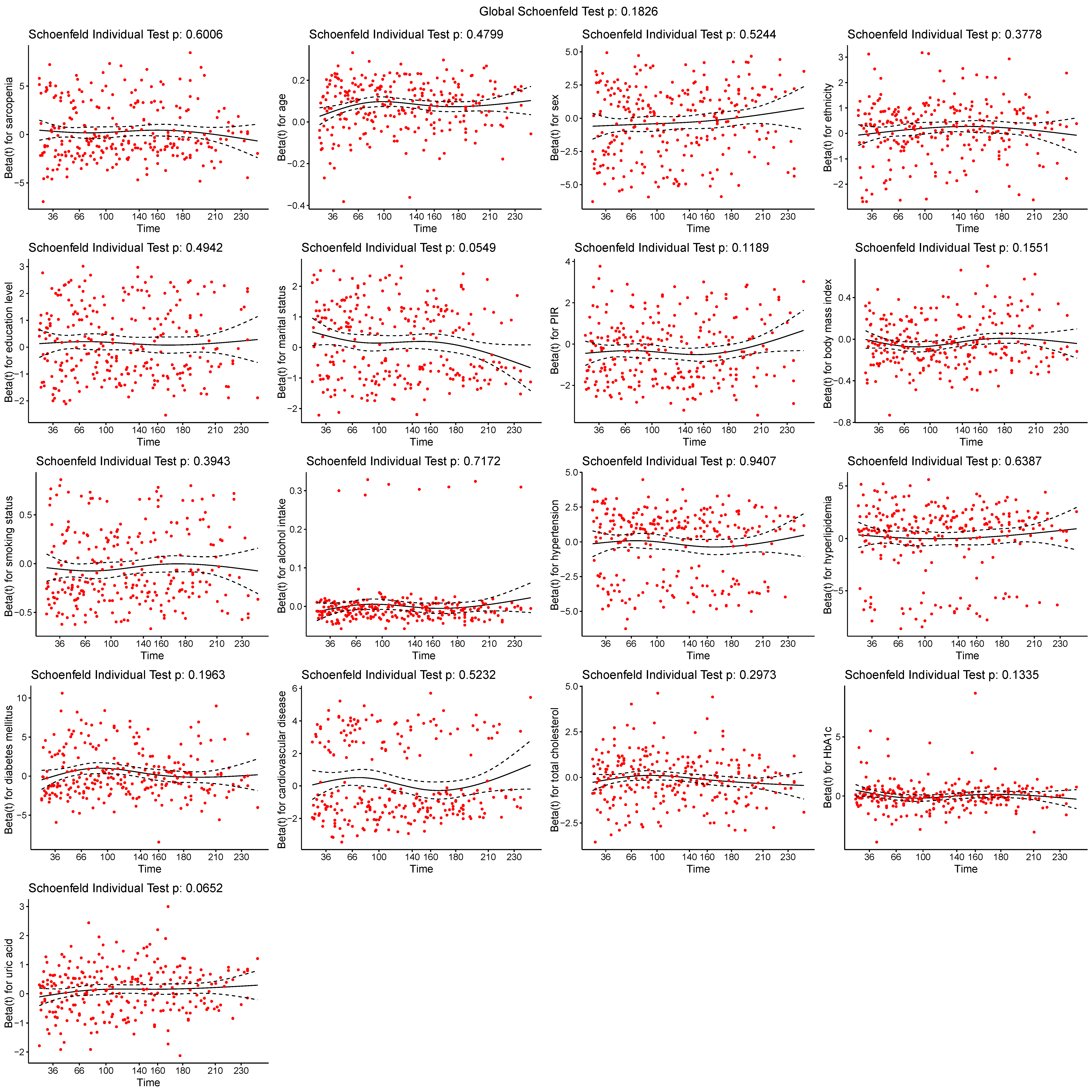

Supplement: Supplementary file 1 [file medi-105-e50000-s001.tif]

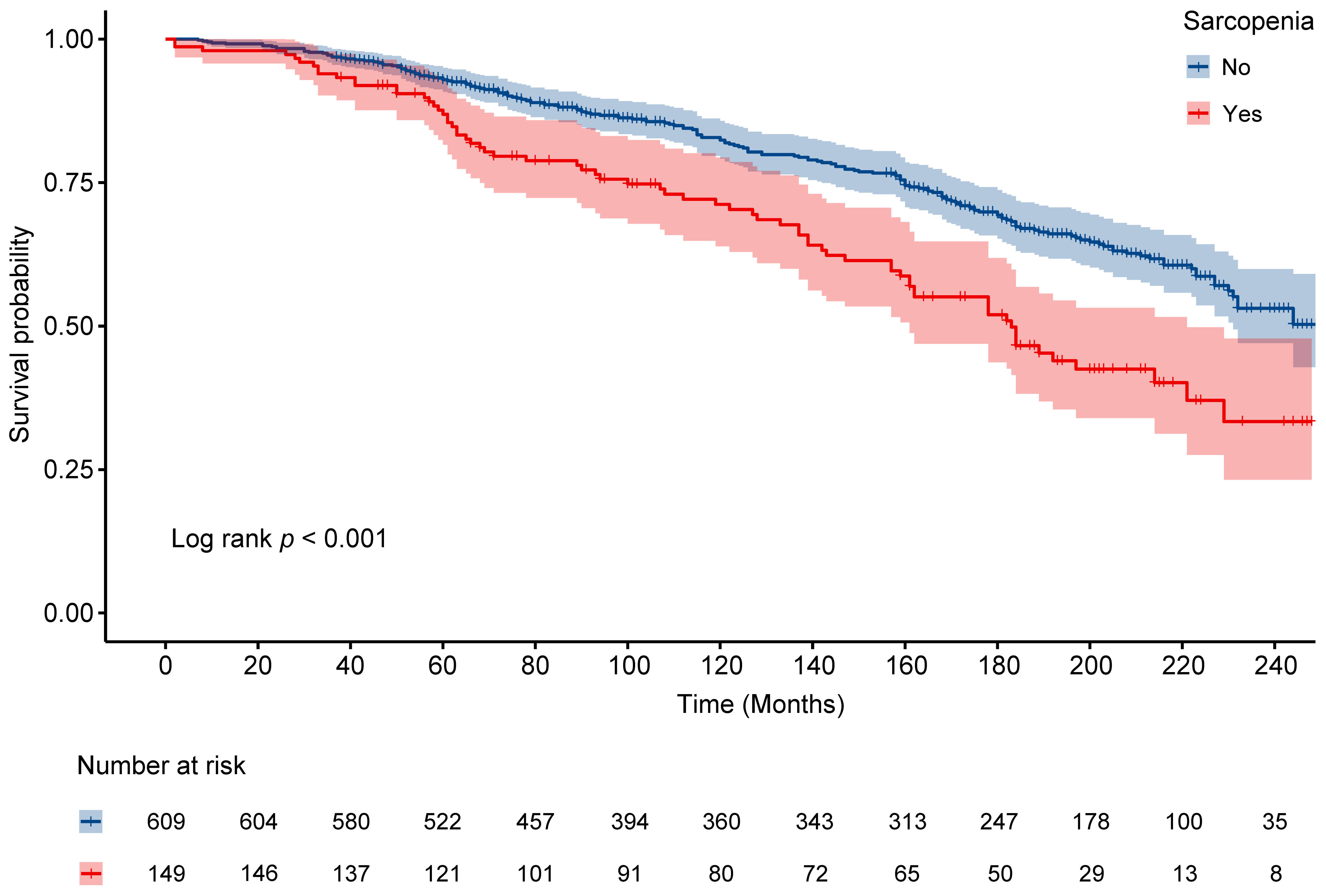

Supplement: Supplementary file 4 [file medi-105-e50000-s004.tif]
