# Supplementary material for: Sarcopenia, an independent predictor for all-cause mortality in rheumatoid arthritis: Insights from the NHANES database
Source: Medicine (Baltimore). 2026 Jul 31;105(31):e50000. doi: 10.1097/MD.0000000000050000 (PMC13433025; doi:10.1097/MD.0000000000050000)
Supplement: Supplementary file 2 [file medi-105-e50000-s002.docx]

| **Supplementary Table 1** Baseline characteristics of participants with complete covariate data | | | | |
| --- | --- | --- | --- | --- |
| **Characteristics** | **Total (n = 758)** | **Sarcopenia** |  | **P value** |
|  |  | **No (n = 609)** | **Yes (n = 149)** |  |
| **Age, mean (SD)** | 56.74 (14.47) | 55.21 (14.29) | 63.00 (13.51) | <0.001 |
| **Sex, n (%)** |  |  |  | 0.988 |
| Male | 315 (41.56) | 253 (41.54) | 62 (41.61) |  |
| Female | 443 (58.44) | 356 (58.46) | 87 (58.39) |  |
| **Ethnicity, n (%)** |  |  |  | <0.001 |
| Mexican American | 145 (19.13) | 83 (13.63) | 62 (41.61) |  |
| Other Hispanic | 48 (6.33) | 37 (6.08) | 11 (7.38) |  |
| Non-Hispanic White | 349 (46.04) | 285 (46.80) | 64 (42.95) |  |
| Non-Hispanic Black | 181 (23.88) | 174 (28.57) | 7 (4.70) |  |
| Other Race | 35 (4.62) | 30 (4.93) | 5 (3.36) |  |
| **Education level, n (%)** |  |  |  | <0.001 |
| Less than high school | 290 (38.26) | 217 (35.63) | 73 (48.99) |  |
| High school or equivalent | 194 (25.59) | 153 (25.12) | 41 (27.52) |  |
| College or above | 274 (36.15) | 239 (39.24) | 35 (23.49) |  |
| **Marital status, n (%)** |  |  |  | 0.042 |
| Married and a partner | 437 (57.65) | 343 (56.32) | 94 (63.09) |  |
| Never married | 55 (7.26) | 51 (8.37) | 4 (2.68) |  |
| Widowed, divorced or separated | 266 (35.09) | 215 (35.30) | 51 (34.23) |  |
| **Poverty to income ratio, n (%)** |  |  |  | 0.025 |
| <1.3 | 278 (36.68) | 215 (35.30) | 63 (42.28) |  |
| 1.3-3.5 | 288 (37.99) | 227 (37.27) | 61 (40.94) |  |
| >3.5 | 192 (25.33) | 167 (27.42) | 25 (16.78) |  |
| **Smoking status, n (%)** |  |  |  | 0.207 |
| Never | 316 (41.69) | 249 (40.89) | 67 (44.97) |  |
| Current | 223 (29.42) | 188 (30.87) | 35 (23.49) |  |
| Former | 219 (28.89) | 172 (28.24) | 47 (31.54) |  |
| **Alcohol intake, n (%)** |  |  |  | 0.052 |
| Never | 344 (45.38) | 269 (44.17) | 75 (50.34) |  |
| Current | 219 (28.89) | 188 (30.87) | 31 (20.81) |  |
| Former | 195 (25.73) | 152 (24.96) | 43 (28.86) |  |
| **Body mass index, n (%)** |  |  |  | <0.001 |
| <25 | 181 (23.88) | 167 (27.42) | 14 (9.40) |  |
| 25-30 | 268 (35.36) | 217 (35.63) | 51 (34.23) |  |
| >30 | 309 (40.77) | 225 (36.95) | 84 (56.38) |  |
| **Hypertension, n (%)** |  |  |  | 0.009 |
| No | 321 (42.35) | 272 (44.66) | 49 (32.89) |  |
| Yes | 437 (57.65) | 337 (55.34) | 100 (67.11) |  |
| **Hyperlipidemia, n (%)** |  |  |  | 0.173 |
| No | 147 (19.39) | 124 (20.36) | 23 (15.44) |  |
| Yes | 611 (80.61) | 485 (79.64) | 126 (84.56) |  |
| **Diabetes mellitus, n (%)** |  |  |  | <0.001 |
| No | 590 (77.84) | 497 (81.61) | 93 (62.42) |  |
| Yes | 168 (22.16) | 112 (18.39) | 56 (37.58) |  |
| **Cardiovascular disease, n (%)** |  |  |  | <0.001 |
| No | 591 (77.97) | 493 (80.95) | 98 (65.77) |  |
| Yes | 167 (22.03) | 116 (19.05) | 51 (34.23) |  |
| **HbA1c, %, mean (SD)** | 5.83 (1.09) | 5.76 (1.06) | 6.12 (1.19) | <0.001 |
| **Total cholesterol, mmol/L, mean (SD)** | 5.21 (1.11) | 5.21 (1.11) | 5.23 (1.15) | 0.832 |
| **Uric acid, mg/dL, mean (SD)** | 5.49 (1.58) | 5.43 (1.59) | 5.70 (1.49) | 0.059 |
| **25-hydroxyvitamin D, (nmol/L), mean (SD)** | 58.72 (22.06) | 59.10 (22.34) | 57.19 (20.93) | 0.394 |
| **Physical activity, MET-min/week, median (IQR)** | 726.07 (246.29-2287.50) | 732.13 (240.00-2268.00) | 720.00 (253.20-2355.00) | 0.415 |

SD, standard deviation; IQR, interquartile range; MET, metabolic equivalent of task.
