# Supplementary material for: Sarcopenia, an independent predictor for all-cause mortality in rheumatoid arthritis: Insights from the NHANES database
Source: Medicine (Baltimore). 2026 Jul 31;105(31):e50000. doi: 10.1097/MD.0000000000050000 (PMC13433025; doi:10.1097/MD.0000000000050000)
Supplement: Supplementary file 3 [file medi-105-e50000-s003.docx]

| **Supplementary Table 2**  Association of sarcopenia with all-cause mortality in RA patients (sensitivity analysis) | | | | | | | |  |
| --- | --- | --- | --- | --- | --- | --- | --- | --- |
| **Group** | **Model 1** |  | **Model 2** |  | **Model 3** |  | |  |
|  | **HR (95% CI)** | **p value** | **HR (95% CI)** | **p value** | **HR (95% CI)** | **p value** | |  |
| Non-sarcopenia | Reference |  | Reference |  | Reference |  | |  |
| Sarcopenia | 1.846 (1.402, 2.430) | < 0.001 | 1.658 (1.221, 2.250) | 0.001 | 1.593 (1.146, 2.215) | 0.006 | |  |
| Model 1: Unadjusted crude model.  Model 2: Adjusted model for age, sex, race/ethnicity, education level, marital status, PIR.  Model 3: Adjusted model incorporating additional adjustments for BMI, smoking, alcohol intake, hypertension, hyperlipidemia, diabetes mellitus, cardiovascular disease, total cholesterol, HbA1c, uric acid RA, rheumatoid arthritis  RA=rheumatoid arthritis  HR=hazard ratio  CI=confidence intervals | | | | | | |  | |
|  |  |  |  |  |  |  |  | |
|  |  |  |  |  |  |  |  | |
|  |  |  |  |  |  |  |  | |
|  |  |  |  |  |  |  |  | |
